# Automated glioblastoma patient classification using hypoxia levels measured through magnetic resonance images

**DOI:** 10.1186/s12868-024-00871-2

**Published:** 2024-05-25

**Authors:** Mohammad Amin Shahram, Hosein Azimian, Bita Abbasi, Zohreh Ganji, Zahra Khandan Khadem-Reza, Elham Khakshour, Hoda Zare

**Affiliations:** 1 Department of Medical Physics, Faculty of Medicine, Mashhad University of Medical Sciences, Mashhad, Iran; 2 Medical Physics Research Center, Mashhad University of Medical Sciences, Mashhad, Iran; 3 Department of Radiology, Faculty of Medicine, Mashhad University of Medical Sciences, Mashhad, Iran

**Keywords:** Glioblastoma, Hypoxia, MRI, PET, Machine learning

## Abstract

**Introduction:**

The challenge of treating Glioblastoma (GBM) tumors is due to various mechanisms that make the tumor resistant to radiation therapy. One of these mechanisms is hypoxia, and therefore, determining the level of hypoxia can improve treatment planning and initial evaluation of its effectiveness in GBM. This study aimed to design an intelligent system to classify glioblastoma patients based on hypoxia levels obtained from magnetic resonance images with the help of an artificial neural network (ANN).

**Material and method:**

MR images and PET measurements were available for this study. MR images were downloaded from the Cancer Imaging Archive (TCIA) database to classify glioblastoma patients based on hypoxia. The images in this database were prepared from 27 patients with glioblastoma on T1W + Gd, T2W-FLAIR, and T2W. Our designed algorithm includes various parts of pre-processing, tumor segmentation, feature extraction from images, and matching these features with quantitative parameters related to hypoxia in PET images. The system’s performance is evaluated by categorizing glioblastoma patients based on hypoxia.

**Results:**

The results of classification with the artificial neural network (ANN) algorithm were as follows: the highest sensitivity, specificity, and accuracy were obtained at 86.71, 85.99 and 83.17%, respectively. The best specificity was related to the T2W-EDEMA image with the tumor to blood ratio (TBR) as a hypoxia parameter. T1W-NECROSIS image with the TBR parameter also showed the highest sensitivity and accuracy.

**Conclusion:**

The results of the present study can be used in clinical procedures before treating glioblastoma patients. Among these treatment approaches, we can mention the radiotherapy treatment design and the prescription of effective drugs for the treatment of hypoxic tumors.

## Introduction

Most patients with neurological disorders who suffer a stroke die from brain tumors, which are masses of abnormal cells in brain tissue [1]. Brain tumors are generally divided into primary and secondary. Primary brain tumors include those tumors that originate from brain tissues or tissues around the brain and are divided into benign and malignant groups [2]. Most malignant central nervous system (CNS) tumors are glioblastoma multiforme (GBM), which accounts for 49.1% of all invasive primary brain tumors. As the most common form of malignant brain tumors, GBM has the highest incidence rate of 3.23 per 100,000 people [3]. . The average survival of patients is usually about 12 to 15 months from the diagnosis time [4]. According to studies, glioblastoma patients have one of the lowest survival rates among all cancers. Less than 30% of newly diagnosed patients survive more than one year, and only 3–5% survive more than two years [5]. More than half a century has passed since the use of chemotherapy drugs along with radiotherapy to treat brain tumors. However, treatment outcomes for brain tumors, especially glioblastoma, are highly variable. Many factors are involved in the ineffectiveness of GBM treatment, like the heterogeneous nature of the tumor, the relatively high age at the onset of the disease, the metastasis of malignant cells to the surrounding areas, and various mechanisms that cause tumor resistance to radiation therapy, especially hypoxia [6].

This section presents the studies that have been done in determining the hypoxia status of brain tumors, especially in glioblastoma patients. These studies were collected and separated into three parts to evaluate the state of tumor hypoxia in patients with the help of oxygen-sensing electrodes, PET imaging, and MRI imaging methods.

Sydney M. Evans et al. (2004) compared the Eppendorf electrode method and the method of using the EF5 kit to measure tumor hypoxia. The results showed that most glial-derived tumor cells in 28 studied cases had mild to moderate hypoxia levels (10–0.5% pO_2_) [7]. Brian E. Lally et al. (2006) measured tissue oxygen pressure using the Eppendorf pO_2_ histograph for 23 glioma patients. They showed that for 13 patients with high-grade glioma and ten patients with low-grade glioma, the average pO_2_ of the tumor for the whole group was 5.1 mm Hg, which indicated a hypoxic condition based on the classification of the study [8]. Alexander M. Spence et al. (2008) presented a study to evaluate the effect of hypoxic tumor volume on tumor resistance to radiation therapy and glioblastoma survival in 22 GBM patients. In patients whose tumors contained Hypoxic Volume (HV) or Maximum Tumor to Blood ratio (T/Bmax) was higher than average, based on Kaplan-Meier curves, survival was at lower levels (HV 12.8 cm3, T/B_max_ 2.06; *P* < 0.0005) [9]. Yamamoto et al. (2012) investigated the level of hypoxia in 30 patients with newly diagnosed glioma tumors using FMISO PET and compared the results with tumor grade. Tumor hypoxia was measured using the amount of radiotracer absorption in PET images, 120 min after drug injection and with the help of TBR_max_ parameter. The results showed that Low-grade gliomas did not have hypoxia, and high-grade gliomas showed hypoxia. The amount of TBRmax FMISO in glioblastoma was significantly higher than grade III gliomas [10]. Elizabeth R. Gerstner et al. (2016) investigated the impact of hypoxia measured by MRI and PET imaging on survival in 50 glioblastoma patients. The results showed that those tumors with the highest baseline markers of hypoxia (F-FMISO SUVpeak) were associated with worse survival [11]. Niha Beig et al. (2018) evaluated radiomic texture descriptors obtained from MRI of 115 glioblastoma patients that can indicate tumor heterogeneity in hypoxic conditions. The results showed that the radiomics features identified in this research could be used to measure hypoxia [12]. Shiliang Huang et al. (2021) investigated the hypoxia features of MRI and PET images in 33 patients with GBM to evaluate tumor drug resistance. There was a positive correlation between hypoxia volume and volume ratio (rs = 0.77, *P* < 0.0001), as well as hypoxia volume and T1-enhancing tumor volume (rs = 0.75, *P* < 0.0001). Bevacizumab-refractory GBM patients exhibit hypoxia as a critical disease biomarker [13]. Ramon F Barajas, Jr. et al. (2022) studied the immunotherapy responses in tumor hypoxia of six glioblastoma patients due to the possible increased incidence of pseudoprogression. Given that hypoxia is an important determinant of glioblastoma regrowth, they hypothesized that [18 F] FMISO PET could provide an additional physiological measure to detect immunotherapy failure. The hypoxic fraction was defined as the ratio of hypoxic volume to T1W gadolinium enhancing volume. Results showed a mean hypoxia rate of 9.8 ± 10% in the four patients with false progression and Two subjects with recurrent tumor showed a mean hypoxic deficit of 131 ± 66%. These results demonstrate that noninvasive assessment of hypoxic fraction by FMISO PET/MRI is clinically feasible and serves as a specific biological metric of treatment failure [14].

From the results of the studies, it can be concluded that most glioblastoma patients have treatment resistance caused by tumor hypoxia. The relationship between the present study and other studies on the importance of measuring hypoxia and predicting the hypoxia status of glioblastoma tumors is to improve the treatment of these patients. There are limitations to checking the hypoxia status of tumors: the invasiveness of the oxygen-sensing electrode method, the non-routine use of PET for glioblastoma patients in clinical procedures, the low spatial resolution of PET, and the lack of access to radiotracers related to determining hypoxia due to their scarcity and high cost. Also, we have limitations in using advanced and functional MRI techniques. These techniques, such as Bold MRI and perfusion techniques, require advanced and expensive devices and equipment that are not available in all places, and it is not possible to perform these protocols for glioblastoma patients routinely during the treatment period. As seen in the studies, a combination of MRI and PET imaging systems is currently used to identify these tumors [11, 13].

In this study, an intelligent system was designed to determine glioblastoma patients’ hypoxia levels based on the radiomics features extracted from structural MRI images, a non-invasive method. For this purpose, lower-cost and more accessible structural MRI protocols have been used for glioblastoma patients. These protocols have been used to overcome the limitations of advanced PET/MRI methods.

## Materials and methods

### Participants

The images for this study were collected from the TCIA (https://www.cancerimagingarchive.net/ ) database [15]. In this study, T1W + Gd, T2W-FLAIR, and T2W MRI images were obtained from glioblastoma patients, all of whom were included in the clinical trial plan (ACRIN 6684). Also, the PET images used in this study were obtained as a 20-minute static 18 F-FMISO PET emission image was acquired 110 min after injection of 3.7 MBq/kg of 18 F-FMISO. The total number of patients in this study was 45. However, since not all patients underwent PET scan and we needed the hypoxia parameters related to this modality, 27 patients were selected for this study. Finally, the MRI images of the patients, along with their clinical information related to the PET scan hypoxia parameters, including maximum tumor-to-background (TBR_max_) and hypoxic tumor volume (HV), were obtained from the TCIA database. As shown in Table 1, the demographics of the study population included their mean age, Karnofsky Performance Score (KPS), and gender. The Karnofsky Performance Status measures the capability of cancer patients to perform ordinary tasks on a scale from 0 to 100. A higher score means the patient can perform daily activities better [16].

**Table 1:** Patient demographics of the study

Features	Number (percentage)
Gender	27
Male	18 (66.7%)
Female	9 (33.3%)
Mean age ± standard deviation	58.70 ± 7.97
Male	57.77 ± 7.68
Female	60.55 ± 8.67
Karnofsky performance status	
Less than 80	18.6%
More than 80	81.4%
Average life expectancy from the time of presentation	412 days

### MRI acquisition

Structural MR images used in this study on three weights, including T2W, T2W-FLAIR, and T1W with injection, were taken using three device models (GE, Siemens, Philips). The complete specifications of the sequences used in the present study are given in Table 2 (The TI value for the FLAIR weight of all scanners is equal to 2.5s).

**Table 2:** MRI Image Acquisition Parameters

Scanner	Weighted	TR(s)	TE(s)	ST(mm)	Matrix Size
GE (1.5T)	T2	3	0.1	5	512*512
	FLAIR	10	0.1	5	256*256
	T1	0.4	0.02	5	256*256
GE (3T)	T2	5	0.12	2	512*512
	FLAIR	10	0.13	5	256*256
	T1	0.5	0.01	5	256*256
Siemens (1.5T)	T2	5	0.1	5	384*512
	FLAIR	10	0.1	5	384*512
	T1	0.5	0.01	5	384*512
Siemens (3T)	T2	3.2	0.4	1	256*256
	FLAIR	10	0.07	5	448*512
	T1	0.19	0.004	5	448*512
Philips (1.5T)	T2	5	0.1	5	512*512
	FLAIR	10	0.14	5	320*320
	T1	0.4	0.02	5	320*320
Philips (3T)	T2	3	0.1	5	560*560
	FLAIR	4.8	0.3	5	432*432
	T1	0.5	0.01	5	432*432

TR: Time of Repetition

TE: Time of Echo

ST: Slice Thickness

### Pre-processing

**Fig. 1:**
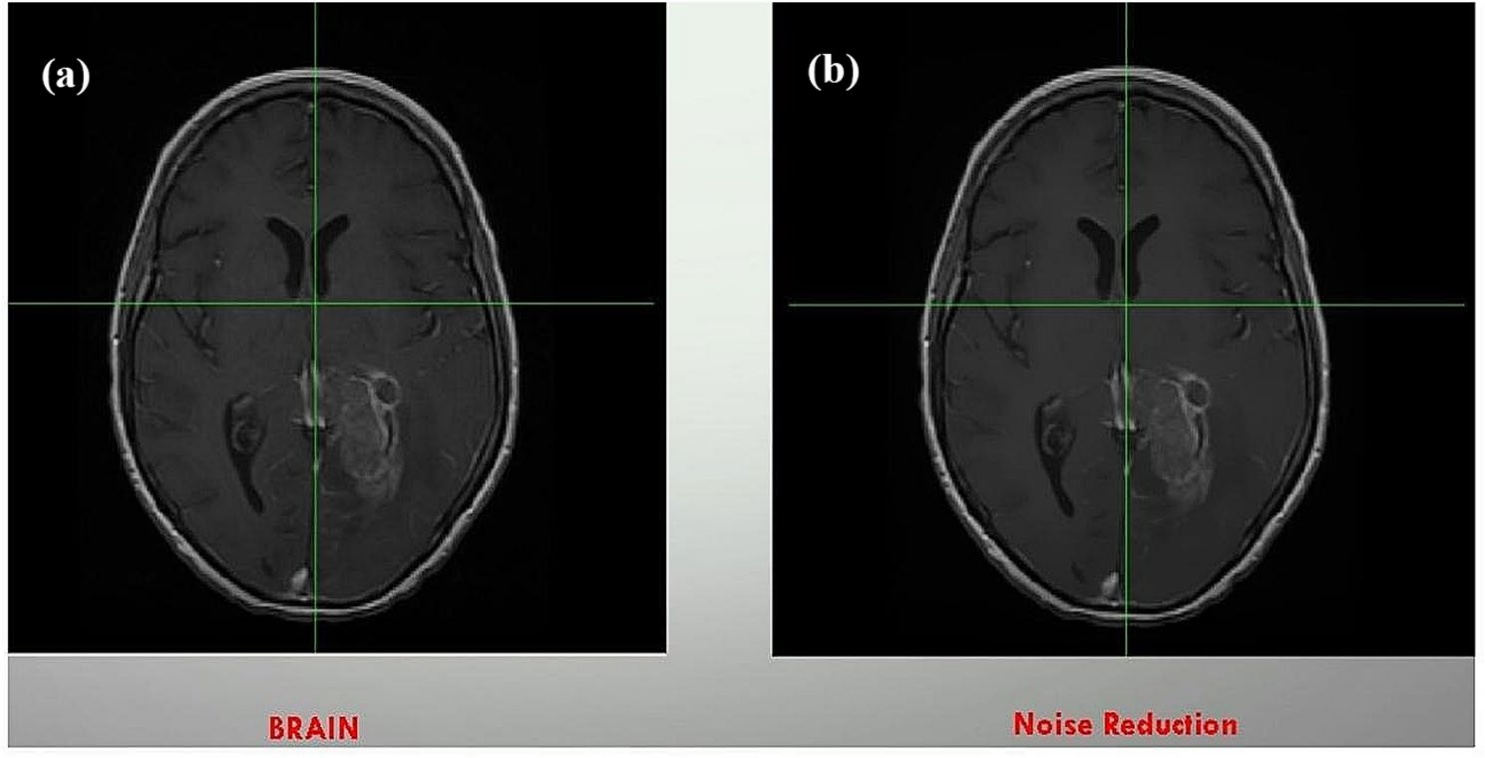
Original brain image (**a**) and noise-reduced image (**b**)

After acquiring MR images, a preprocessing step is required to prepare the images for segmentation. In this study, FSLv6.0 software was used to reduce the noise of the images by using the SUSAN noise reduction tool with the threshold by default. At this stage, the skull was not removed because parts of the tumors were located at the periphery of the brain, and if the skull was removed, part of the tumor would be removed from the images and related features would be lost. The original brain tumor image and the noise-reduced image after the preprocessing step are shown in Fig. 1.

### Manual segmentation

**Fig. 2:**
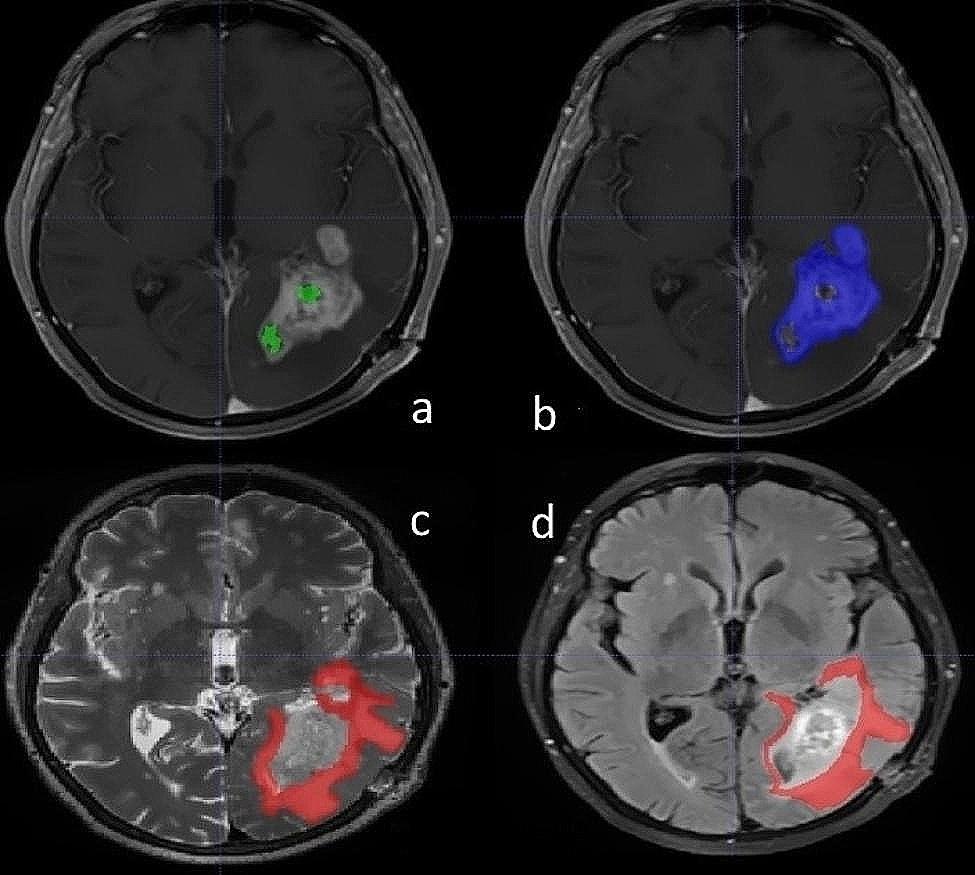
Tumor segmentation via ITK-SNAP by using input (**a**) T1W + GD image for tumor necrosis area, (**b**) T1W + GD image for enhancement area, (**c**) T2W image for edema area, and (**d**) T2W-FLAIR for edema area

In this study, the images were manually segmented into four areas by an experienced radiologist using the ITK-SNAP software. In such a way that in each relevant slice, the region of interest (ROI) was drawn, and finally, the volume of the tumor was segmented. Then these areas were examined by a neurosurgeon and the following measures were taken after his approval. These four areas included: the tumor necrosis area in T1W images with injection (24 regions), the tumor enhancement area in T1W images with injection (27 regions), the tumor edema area in T2W images (24 regions), and the tumor edema area in T2W-FLAIR images (26 regions). Figure 2 shows the segmented regions in ITK-SNAP software.

.

### Feature extraction

Pyradiomics, an image analysis library installed in Python software, was used to extract radiomic features from segmented regions of the tumor [17]. Twelve shape-dependent features, 158 First-order features (histogram features), and 612 s-order features (texture features) were extracted from the segmented area in the previous step. The first-order features show the intensity distribution of the gray levels of the voxels in the region of interest (ROI), including entropy, energy, and skewness. Second-order features describe the spatial relationship of gray level values between different voxels using different algorithms [18]. The number of slices containing segmented tumor regions for each imaging protocol and region of the image is given in Table 3.

**Table 3:** Number of slices of segmented tumor regions for imaging protocol

Scanner Type	Image Protocol	Region	Number of Slices
GE 1.5T (30 slices) GE 3T (98 slices) Siemens 1.5T (21 slices) Siemens 3T (92 slices) Philips 1.5T (46 slices) Philips 3T (48 slices)	T2	Edema	335
GE 1.5T (34 slices) GE 3T (54 slices) Siemens 1.5T (42 slices) Siemens 3T (18 slices) Philips 1.5T (45 slices) Philips 3T (83 slices)	T2-FLAIR	Edema	276
GE 1.5T (4 slices) GE 3T (23 slices) Siemens 1.5T (25 slices) Siemens 3T (8 slices) Philips 1.5T (27 slices) Philips 3T (19 slices)	T1	Necrosis	106
GE 1.5T (13 slices) GE 3T) 45 slices ( Siemens 1.5T (38 slices) Siemens 3T (15 slices) Philips 1.5T (32 slices) Philips 3T (35 slices)	T1	Enhance	178

### Features harmonization with combat method

With the increase of multi-scanner studies, non-biological variations introduced by different image access settings, affect the reliability and reproducibility of radiomic results. for this reason there’s a greater need for addressing non-biological variance presented by differences in MRI scanners and acquisition protocols [19]. As a means of reducing the inherent variability in medical images, harmonization processes have been proposed. Harmonization aims to overcome the lack of reproducibility in radiomics features and comparability among medical images [20]. In order to eliminate the effects of the scanner and increase the repeatability of radiomic features in brain MRI images, at this stage, combat harmonization methods on radiomic features were utilized. The combat method was used to apply the field strength of each scanner as a batch effect to the radiomics features, resulting in harmonized features. The code is available at the following address: https://github.com/Jfortin1/ComBatHarmonization. Additionally, prior to the classifications and following this step, the z-score method was utilized to normalize the features [21, 22].

### Classification

After the feature harmonization and normalization stages, the automatic classification of the patient’s tumor hypoxia status in 4 sections: edema (T2W), edema (FLAIR), necrosis and enhancement (T1W), and with the help of artificial neural network (ANN) were performed in MATLAB software. Artificial neural networks (ANNs) are computational models based on predefined activation functions that receive inputs and deliver outputs [23]. To deal with the problem of overfitting, we used dropout and early stopping techniques.

**Fig. 3:**
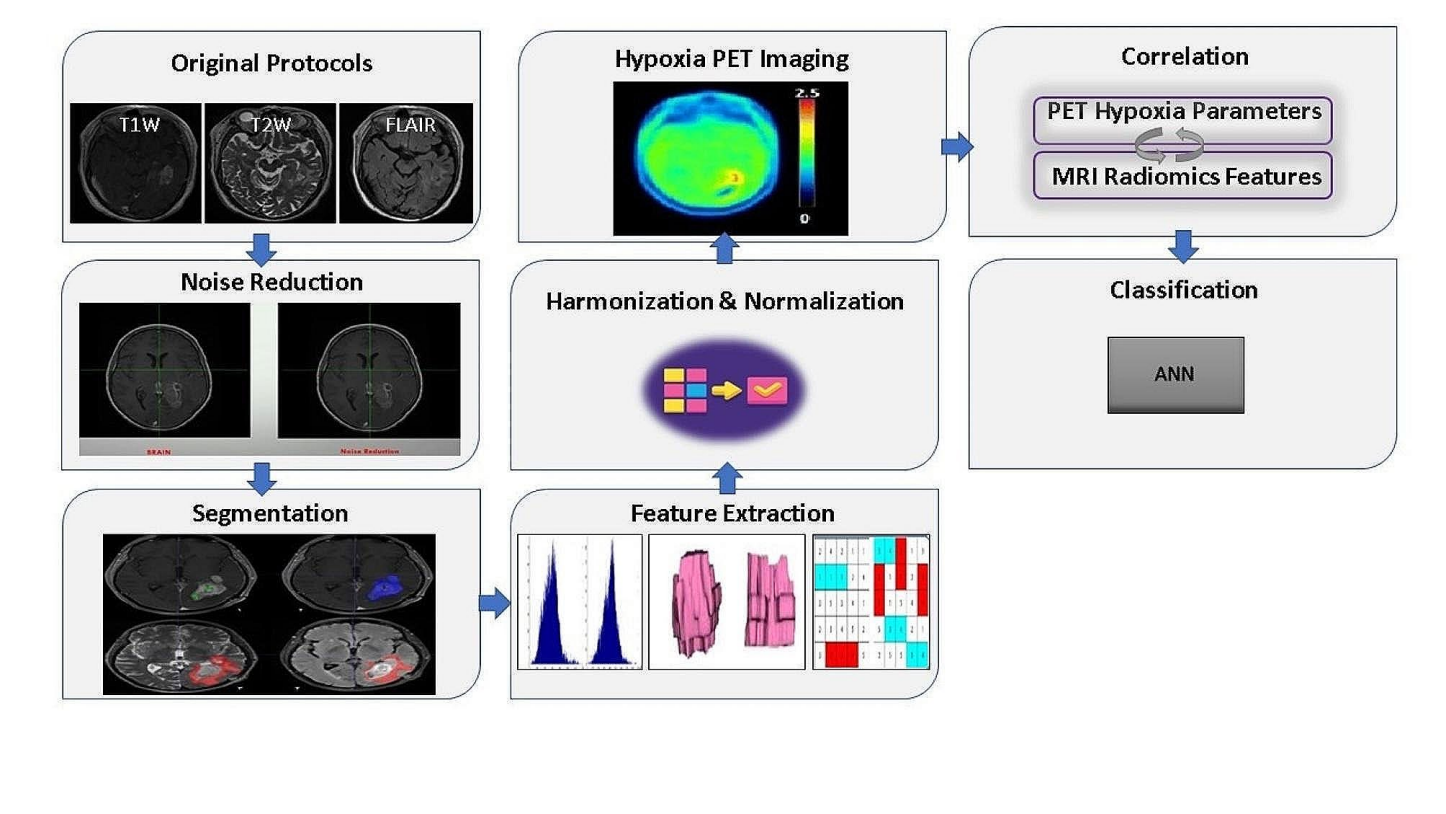
illustrates an overview of the proposed study

Figure 3. *Overview of the methodology and overall workflow.*

## Results

### Evaluation metrics

**Fig. 4:**
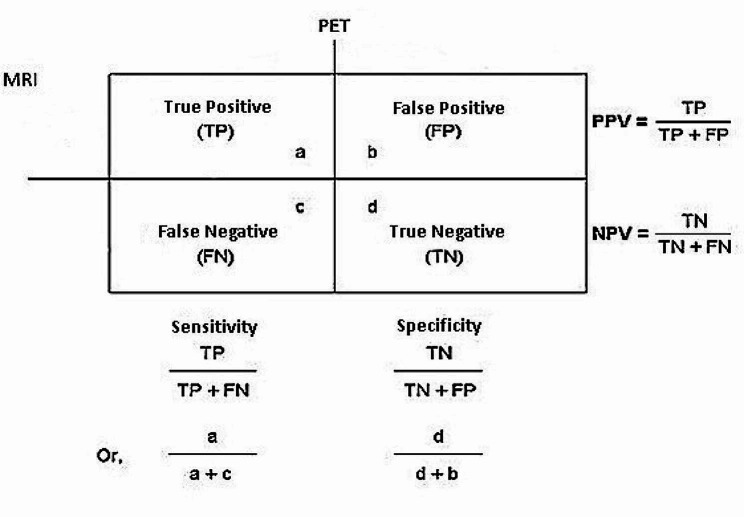
Evaluation metrics for classification model

During this section, evaluation criteria were discussed to be applied when evaluating the effectiveness of the classification algorithm. Also, three statistical parameters of sensitivity, specificity, and accuracy have been used to evaluate the performance of the ANN algorithm. Before we go into different evaluation metrics, we explain some basic terminologies with the help of a confusion matrix (Fig. 4). Finally, using the formulas written in Fig. 4, the system’s sensitivity, specificity, and accuracy were measured.

- TP = The true positive parameter in this article refers to cases that show high hypoxia in the parameters of PET images and high hypoxia in the features of MRI images.
- FP = The false positive parameter in this article represents the cases that show low hypoxia in the parameters of PET images and high hypoxia in the features of MRI images.
- TN = The true negative parameter in this article represents the cases that show low hypoxia in the parameters of PET images and low hypoxia in the features of MRI images.
- FN = The false negative parameter in this article refers to the items that show high hypoxia in the parameters of PET images and low hypoxia in the features of MRI images.

### Evaluation of the ANN algorithm for the classification of hypoxia status

In this classification method, a feed-forward neural network was used to separate the hypoxia regions into low and high classes, which include input, output, and hidden layers. The number of neurons in the input layer was equal to the number of features of the resulting matrix, and the number of neurons in the hidden layer was considered 10. Conversely, the neural network’s output includes one neuron, so it has 0 or 1 output. Output equal to 1 indicates areas with high hypoxia, and output equal to 0 corresponds to images with low hypoxia. 70% of the data was used for training the system and 30% of the data was used for testing and validation. After determining the number of layers and neurons, the ANN classifier was trained on the selected features. The sensitivity, specificity, and accuracy values in the ANN classification were calculated after 30 epochs. The mean results obtained for each protocol and the PET parameters are given in Table 4.

**Table 4:** Evaluation of ANN classification performance

MRI Protocol & PET Parameter	Sensitivity(%)	Specificity(%)	Accuracy(%)
FLAIR – EDEMA (HV)	83.60	79.28	81.51
FLAIR – EDEMA (TBR)	83.98	81.88	81.74
T2 – EDEMA (HV)	**80.19**	**81.15**	**81.41**
T2 – EDEMA (TBR)	**80.34**	**85.99**	**82.07**
T1 – ENHANCE (HV)	**78.77**	**73.54**	**73.66**
T1 – ENHANCE (TBR)	**79.01**	**79.65**	**79.35**
T1 – NECROSIS (HV)	**83.68**	**75.22**	**79.59**
T1 – NECROSIS (TBR)	**86.71**	**80.29**	**83.17**

For each protocol, the highest sensitivity, specificity and accuracy are bolded in the table

## Discussion

The evaluation parameters related to the classification of the hypoxia status based on MRI.

protocols and PET parameters are discussed in this section.

**Fig. 5:**
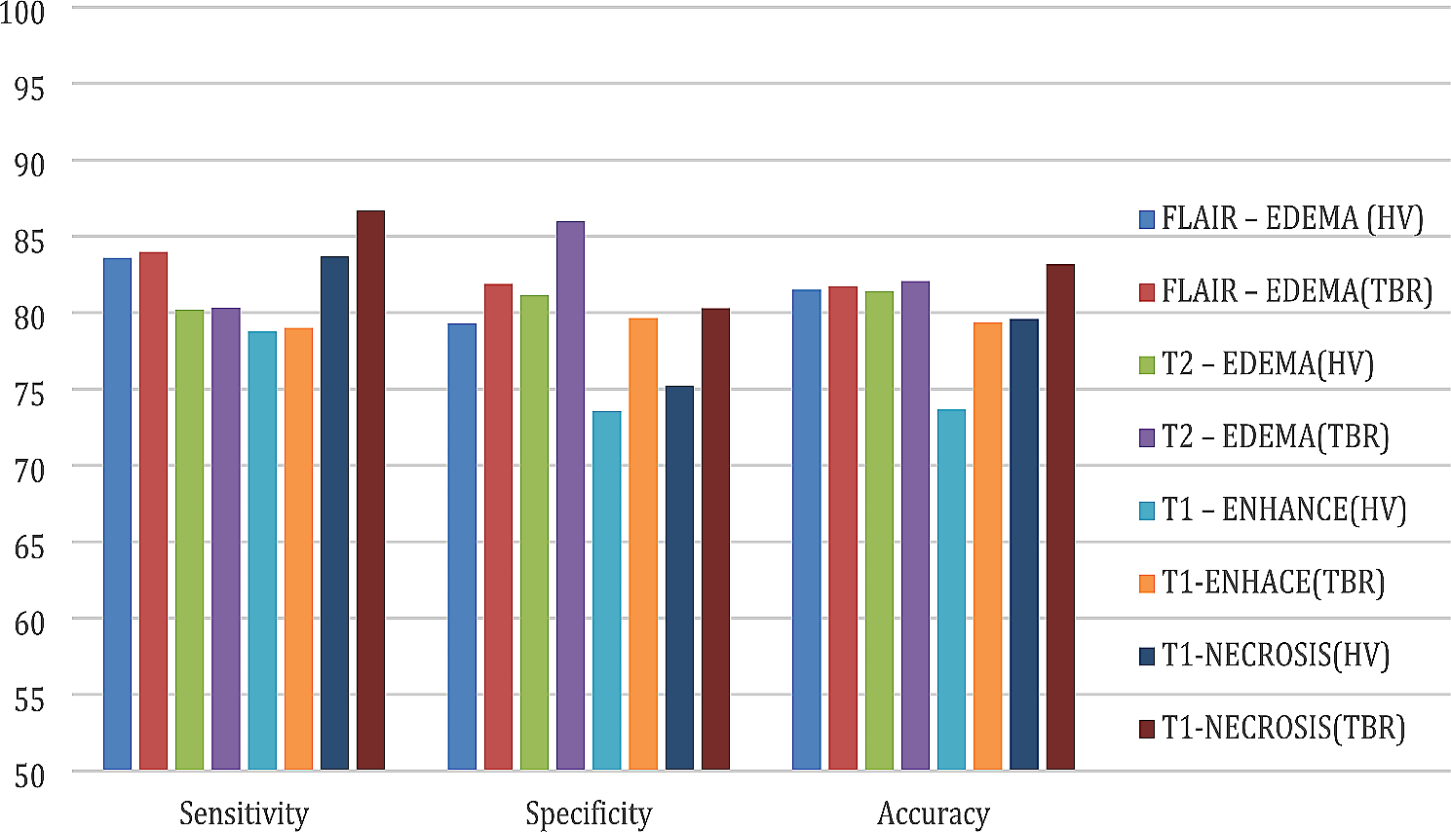
Evaluation parameters of classification for MRI protocols and PET parameters

According to Fig. 5, the highest specificity obtained to classify the hypoxia status of the patient’s tumor in the T2W-EDEMA protocol and with the TBR hypoxia parameter was 85.99%. Using this MRI imaging protocol and PET hypoxia parameter, the highest fraction of patients with low hypoxia in PET, who also have low hypoxia in MRI, can be detected. This may be due to the fact that prolonged exposure to hypoxic conditions can lead to the development of cerebral edema and T2w abnormalities are known to capture proliferative tumor margins and vasogenic edema [12, 24–26]. The highest level of sensitivity was related to the T1W-NECROSIS protocol with the TBR hypoxia parameter, which equals 86.71%. Using this MRI protocol and PET hypoxia parameter, it was possible to detect a fraction of patients with high hypoxia in PET who also have high hypoxia in MRI. It may be because necrosis appears relatively hypointense in Gd-T1W images, especially in the central region of the tumor [12]. The highest level of accuracy was 83.17%, corresponding to the T1W-NECROSIS protocol with the TBR hypoxia parameter, which is the same as the fraction of people whose hypoxia status is the same in MRI and PET (both low or both high).

According to the obtained results, if the patients have all three protocols in the treatment process, it is better to use the T1W-NEC protocol to identify hypoxia. Still, if it was impossible to perform the T1W protocol for some patients, the T2W protocol can be used to determine hypoxia status.

The present study’s results contradict the study of Elizabeth R. Gerstner et al. (2016) [11]. Their results showed that the correlation between most pairs of MRI and PET markers was not statistically significant, and there was only a moderate positive correlation between nCBF and HV. However, in the present study, there was a significant relationship between the radiomic features of the patient’s MRI images and their PET hypoxia parameters. The difference between the present study and this study could be the difference in the extracted features, the type of MRI imaging protocol, and the classification algorithms.

The present study also confirms the study by Niha Beig et al. (2018) [12], which had differences in the number of patients, type of classification algorithms, and extracted features. Also, their gold standard for tumor hypoxia status was gene expression, but in the present study, it was PET parameters. However, both studies proved a significant relationship between hypoxic status and the features of MRI images.

The present study could use conventional MRI images of T1W + Gd, FLAIR, and T2W to nominate affected tumor areas with high or low hypoxia levels. Such results can be used clinically before treating these patients. Among these treatment approaches, we can mention the design of radiotherapy treatment specifically for each group of patients. Also, by knowing the hypoxia condition of each group of patients, different drugs can be used that inhibit the oxidative phosphorylation mechanism (OXPHOS) in the mitochondria. These drugs can reduce oxygen consumption by inhibiting OXPHOS by targeting the electron transfer chains in the mitochondria, and as a result, more oxygen will be available around the tumor tissue. Reoxygenation of radioresistant tumors increases their radiosensitivity and increases tumor cell death as a result of radiotherapy. Among these drugs, Atovaquone, Ivermectin, and Mefloquine can be mentioned.

This study had limitations, such as the lack of access to many patients and the lack of direct participation. Although the overall performance of classifiers depends on the extent to which a dataset represents the original distribution rather than its size. However, classifiers cannot effectively learn with small data sets due to various issues such as overfitting, noise, outliers, and sampling bias, which can render the learned model ineffective. The larger the sample size, the more the results can be generalized. Also, it was not possible to perform deep learning methods in this study due to the limited number of patients. Another limitation was the impossibility of removing the skull due to the tumor’s location at the edges of the brain, which led to the impossibility of using automatic segmentation methods in this study. It is suggested that the present study be conducted with more patients, other classification algorithms, and other hypoxia parameters of PET scans (for example SUV_max_) and other MRI protocols such as dynamic susceptibility contrast (DSC) and dynamic contrast enhancement (DCE). Looking into the future, it is possible to investigate the relationship between the state of tumor hypoxia obtained by analysis of images using advanced MRI techniques with clinical data such as survival, predicting the state of response to treatment, tumor recurrence, and its relationship with the molecular characteristics such as IDH and EGFR status.

## Conclusion

In the present study, the hypoxia status of 27 patients was analyzed using extracted features from MRI images and with the help of PET hypoxia parameter as the gold standard. Using the ANN algorithm, the hypoxia status of patients was classified into two groups, including low and high. The highest average sensitivity, specificity, and accuracy were obtained in the best conditions at 86.71%, 85.99%, and 83.17%, respectively. The results of the present study can be used in clinical measures before treating these patients. Among these treatment approaches, we can mention the design of treatment in radiotherapy and the prescription of effective drugs for tumor treatment, specifically for each group of patients.

## Data Availability

Patient data were collected from the TCIA database and used in the study. Raw data can be downloaded from the TCIA database.https://www.cancerimagingarchive.net/.
